# Clinical burden of pneumococcal septicaemia

**DOI:** 10.1186/1471-2458-14-S1-P13

**Published:** 2014-01-29

**Authors:** Nimetcan Mehmet Orhun, Maihebureti Abuduli, Azam Rahimi, Zafar Ahmad, Zaleha Mh Isa, Syed Mohamed Aljunid

**Affiliations:** 1 UNU-IIGH Building, UKM Medical Center, Jalan Yaacob Latiff 56000 Cheras, Kuala Lumpur, Malaysia; 2 Jabatan Kesihatan Masyarakat, Pusat Perubatan Universiti Kebangsaan Malaysia, Jalan Yaacob Latif, Bandar Tun Razak, 56000 Cheras, Kuala Lumpur, Malaysia

## Background

Streptococcus pneumoniae is a major cause of both mild and severe infections worldwide. The primary clinical syndromes associated with pneumococcal infections are pneumonia, meningitis, bloodstream infections and acute otitis media. Disease rates are highest in children <5 years of age, are low in older children and healthy young adults, and increase again in the elderly. The vast majority of its victims come from developing countries. However there is no data on clinical burden of pneumococcal septicaemia (PS). The aim of this study was to estimate the annual clinical burden of PS in Malaysia.

## Materials and methods

A retrospective review of in-patient medical records with PS (A40.3, A40.9) was conducted at four hospitals in Malaysia from three different regions starting from 1 January 2008 to 31 December 2009. The catchment population was imputed based on each hospital’s location including total local population of hospital’s area plus 30% of state population. Local expert’s opinion was used to obtain outpatient burden of disease. A model characterising rate of PS in Malaysia was developed to estimate the expected annual clinical burden of the disease.

## Results

The total burden of PS was estimated as 17,776 cases, out of this 6% (1,051) were inpatients, while 94% (16,725) were outpatients. Out of total number of cases, 10% (1,858) were paediatric cases and 90% (15,917) were adult cases.

## Conclusions

The burden of PS was higher among adult populations. This disease burden can potentially be reduced by preventive measures such as vaccination.

